# Divergent long-term impacts of lethally toxic cane toads (*Rhinella marina*) on two species of apex predators (monitor lizards, *Varanus* spp.)

**DOI:** 10.1371/journal.pone.0254032

**Published:** 2021-07-22

**Authors:** Lachlan Pettit, Mathew S. Crowther, Georgia Ward-Fear, Richard Shine

**Affiliations:** 1 School of Life and Environmental Sciences, University of Sydney, Sydney, NSW, Australia; 2 Department of Biological Sciences, Macquarie University, Sydney, NSW, Australia; University of Southern Queensland, AUSTRALIA

## Abstract

Biological invasions can massively disrupt ecosystems, but evolutionary and ecological adjustments may modify the magnitude of that impact through time. Such post-colonisation shifts can change priorities for management. We quantified the abundance of two species of giant monitor lizards, and of the availability of their mammalian prey, across 45 sites distributed across the entire invasion trajectory of the cane toad (*Rhinella marina*) in Australia. One varanid species (*Varanus panoptes* from tropical Australia) showed dramatic population collapse with toad invasion, with no sign of recovery at most (but not all) sites that toads had occupied for up to 80 years. In contrast, abundance of the other species (*Varanus varius* from eastern-coastal Australia) was largely unaffected by toad invasion. That difference might reflect availability of alternative food sources in eastern-coastal areas, perhaps exacerbated by the widespread prior collapse of populations of small mammals across tropical (but not eastern) Australia. According to this hypothesis, the impact of cane toads on apex predators has been exacerbated and prolonged by a scarcity of alternative prey. More generally, multiple anthropogenically-induced changes to natural ecosystems may have synergistic effects, intensifying the impacts beyond that expected from either threat in isolation.

## Introduction

Invasive species rank among the greatest threats to biodiversity, and have been implicated in many cases of decline and extinction of native taxa [1, 2]. The need to understand those threats has stimulated extensive research on the impacts of biological invasions, but the majority of those studies have focused on short-term impacts [3]. However, growing evidence documents rapid evolutionary changes both in invaders and in native taxa following their initial contact, such that the impact of the invader may shift through time [4, 5]. Also, the arrival of an invader may stimulate ecological changes that ramify through trophic webs, resulting in phenomena such as mesopredator release [6] and increased abundance of native taxa capable of exploiting opportunities presented by the invader [7, 8]. This effect can be amplified if the invader exerts disproportionally large impacts on certain functional groups within the system (e.g., predator guilds). As a result, the ecological impact of an invasion can change through time. In some cases, the negative impacts of an invasion will be short-lived [9]; but in other cases, populations of an imperilled taxon continue to decline post-invasion, eventually resulting in extinction [10, 11].

Similarly, drivers of ecological change rarely occur in isolation [12]. Impacts of processes such as climate change, habitat degradation, and pollution can interact with invasive species to modify the direction, magnitude or duration of invader impact. Accordingly, we need to assess invader impacts in the context of other changes that interact synergistically or antagonistically over time [13], or affect ecosystem or species resilience [14]. For example, a warming climate may present new opportunities for non-native species to invade areas that were previously outside their thermal tolerances, increasing their impact [15].

A scarcity of research on long-term impacts of invasion, and on synergies among multiple concurrent threats, means that we have little empirical basis from which to allocate priorities for management. If invader impacts are expected to be brief, managers may allocate fewer resources to the problem; whereas if impacts are maintained or increase through time post-colonisation, managers need to take an active role in ameliorating those negative consequences [3]. If invader impacts depend upon some other change–such as the concurrent arrival of another invader–then managers can potentially address the impacts of one alien species by controlling another.

In the present study, we provide data from a large-scale sampling of apex predator populations vulnerable to invasion by a toxic amphibian. Short-term studies have reported catastrophic population declines in larger species of the lizard genus *Varanus* immediately following the arrival of cane toads (*Rhinella marina*) [16–19], but subsequent trajectories of predator populations remain unclear. Some of the varanid species that decline after toad invasion have been inferred to recover, based upon their persistence in sites where cane toads have been present for many decades [20, 21]. Broadly, we predict the impact of toads on varanid abundance to be highest at the invasion front, and for populations to recover over time. However, the timescale and magnitude of this apparent recovery have not been documented. Our extensive sampling provides evidence on these issues.

## Materials and methods

### Study species

Cane toads (*Rhinella marina*) are large anurans (snout-urostyle length up to > 230 mm, mass up to > 500 g) native to South America [22]. Introduced to Australia in 1935 to control pests of sugar cane crops, the cane toad has rapidly spread to occupy over 1.2 million square km [23]. The defensive toxins of cane toads (bufodienolides) are highly poisonous to many of Australia’s frog-eating native predators, and dramatic declines in populations of several such species have occurred at the toad invasion front [24]. We compared the population resilience of two varanid apex predators for whom cane toads pose a threat. Both varanids (“goannas” or “monitor lizards” in vernacular terms) are among the largest terrestrial reptiles in their respective ecosystems.

**Fig 1:**
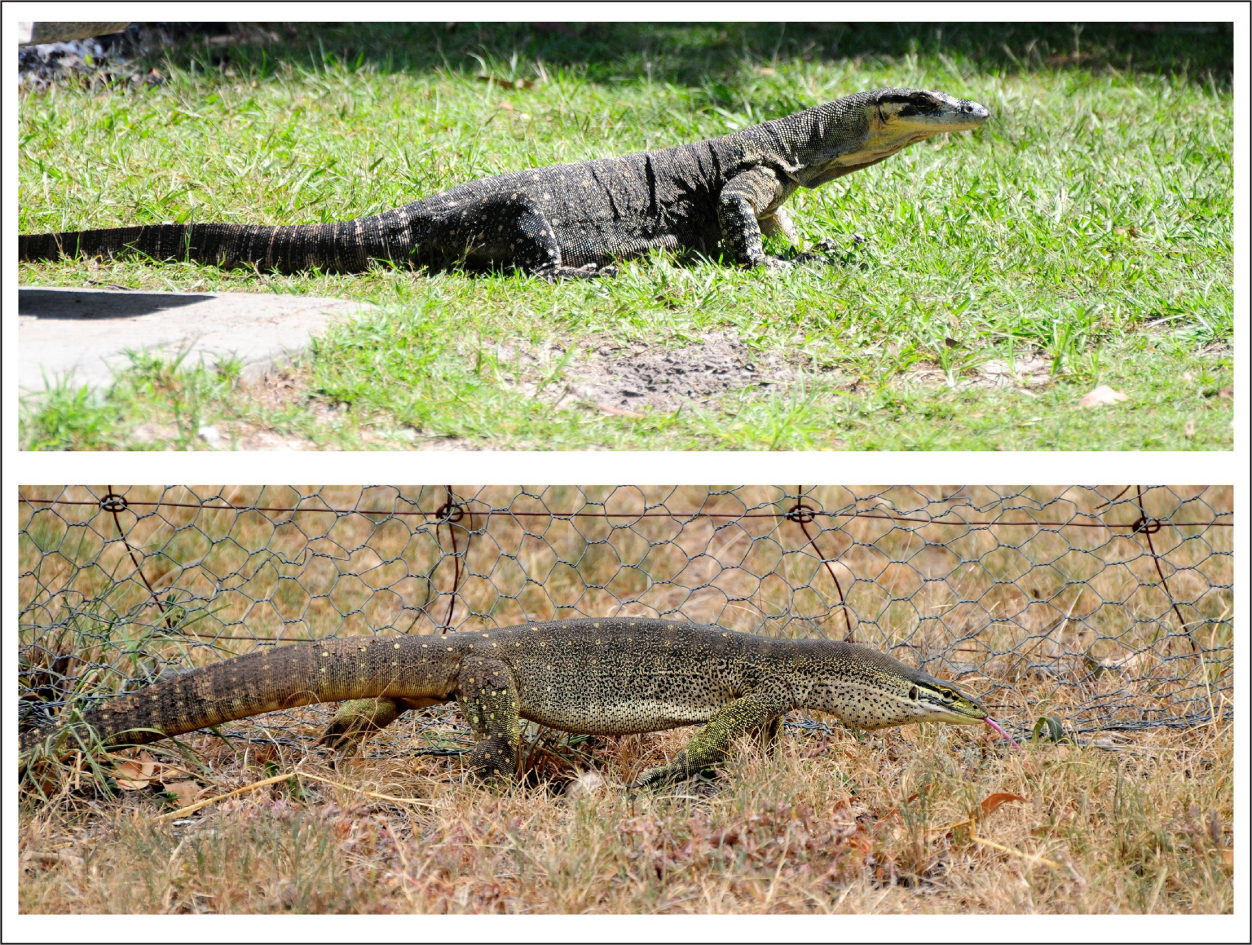
Lace monitor (*Varanus varius*, top panel) and yellow-spotted monitor (*V*. *panoptes*, bottom panel). Both species are large apex predators with generalist diets.

The lace monitor (*Varanus varius*; Fig 1, top panel) is found along the eastern coast of Australia [25], and can grow to 14 kg and exceed 2 metres in total length. These lizards typically inhabit forested areas, and are semi-arboreal [26, 27]. Lace monitors shift the composition of their diet seasonally [28] and often consume small mammals and carrion [28, 29]. Numbers of lace monitors in northern New South Wales reportedly decrease in areas invaded by cane toads [16, 30], but the species is common in some sites where toads have been present for decades [31].

Yellow-spotted monitors (*Varanus panoptes*, Fig 1, lower panel) can grow to 7 kg and up to 2 metres, and are widely distributed through the wet/dry tropics of northern Australia [32]. The species is most abundant on floodplains and along the margins of watercourses that penetrate into drier areas [33]. Like lace monitors, yellow-spotted monitors have a broad diet which shifts with seasonal resource availability [33]. Small mammals (e.g., *Rattus colletti* and *R*. *tunneyi*) are an important component of the diet [34, 35]. Populations of yellow-spotted monitors decline by more than 90% following toad arrival [17–19]. The success of conditioned taste aversion (discouraging consumption of toads by yellow-spotted monitors) in buffering that impact [36] confirms a causal connection between toad invasion and varanid population collapse. Accordingly, yellow-spotted monitors now have a listing of ‘Vulnerable’ across some jurisdictions (e.g., Northern Territory). Nonetheless, yellow-spotted monitors are common in some toad-colonised sites close to the areas where toads were first released [21]. No previous studies have quantified patterns of varanid abundance over the long timeframe of toad occupation in Australia, although there are several studies with data on short-term trajectories of varanid abundance after toad invasion [19, 37].

The two varanid species are broadly similar in body sizes and general morphology (Fig 1) as well as in their general biology, including broad diet [38]. The most obvious difference between the two taxa lies in the habitats they occupy, in turn linked to climatic conditions over their distributions. The lace monitor inhabits forested areas, where ambient temperatures change seasonally and precipitation ranges from relatively aseasonal (southern regions) to seasonal (tropical regions). In contrast, in the areas where it is sympatric with toads the yellow-spotted monitor inhabits a monsoonal climate where daily maximum air temperatures remain high year-round but precipitation is concentrated in a brief “wet season” [34, 39]. Yellow-spotted monitors typically are found in relatively open (often, treeless) tropical floodplains, but also exploit other open habitats such as ocean beaches where they forage for the eggs of sea turtles [40, 41]. The two lizard species differ in habitat use even in areas where they are broadly sympatric, with lace monitors in forested sites and yellow-spotted monitors in more open areas [20].

### Study sites

**Fig 2:**
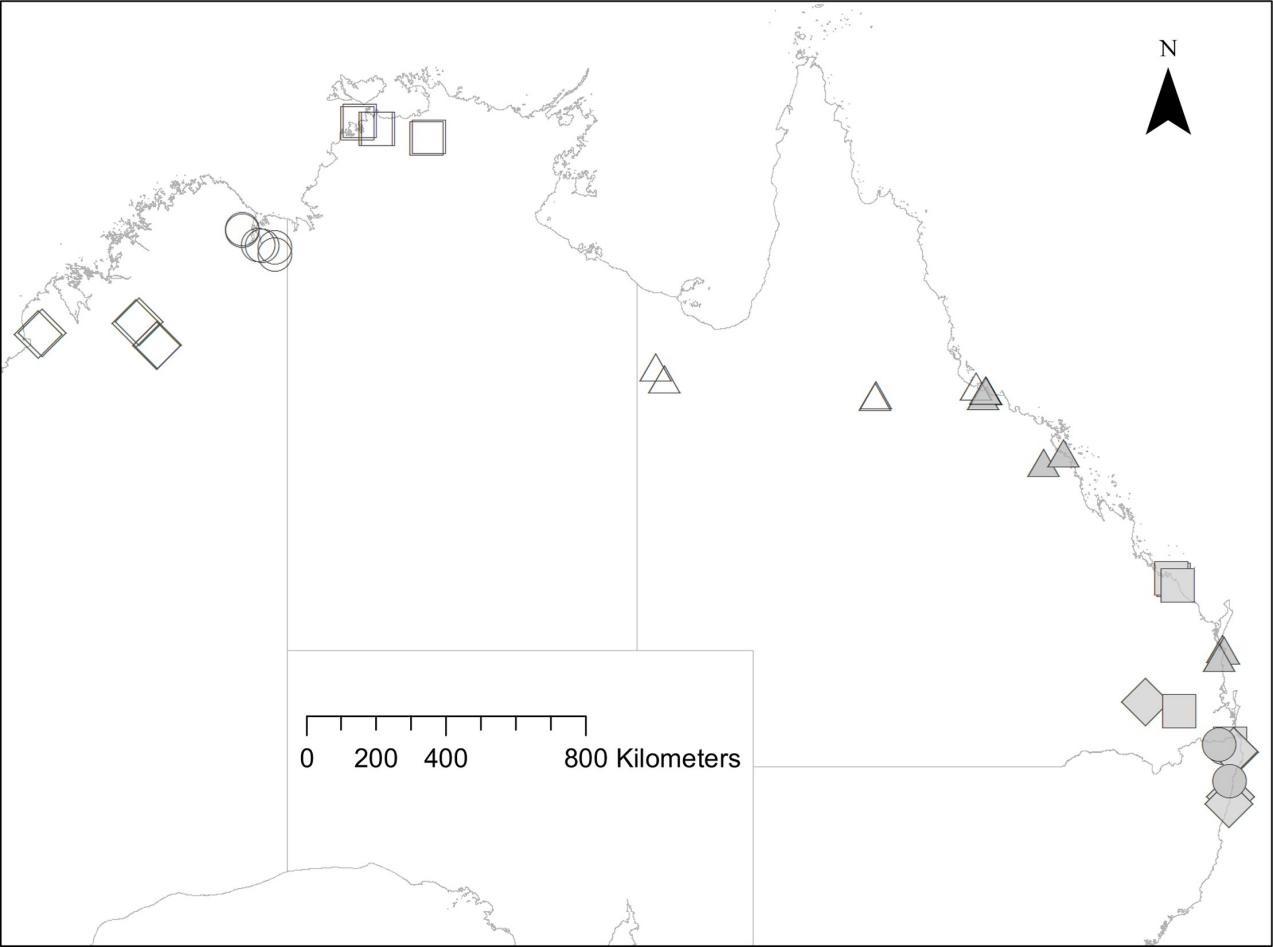
Active searches and motion-triggered cameras were used to estimate the relative abundance of lace monitors (*Varanus varius*) in 21 populations along the east coast of Australia (shaded symbols) and in 24 populations of yellow-spotted monitors (*V*. *panoptes*) in tropical Australia (open symbols). Sites were allocated into invasion stages based on the number of varanid generations that cane toads had been present. Diamonds–uninvaded; circles–recently invaded (1–12 varanid generations); squares–mid-term invaded (13–29 varanid generations); triangles–long-term invaded (30–80 varanid generations). This map was generated in QGIS 3.12.3.

We established two continent-scale transects to evaluate how populations of these two varanid species have responded to cane toads over long timescales (Fig 2). To assess relative abundance, lace monitors were surveyed at 21 sites along the east coast of Australia (the “east coast transect”) between October 2017 and April 2018, and yellow-spotted monitors were surveyed at 24 sites across tropical Australia (the “tropical transect”) between January and May 2019. All sites had contemporary or historical records of the target varanid species, as obtained from literature searches [19, 21, 30, 40, 42–45] and occurrence records from the Atlas of Living Australia database (http://www.ala.org.au - accessed 2017–18).

### Site selection and estimations of cane toad invasion chronology

We selected sites based on the known contemporary presence of either lace monitors (*V*. *varius*) or yellow-spotted monitors (*V*. *panoptes*) derived from the literature, and anecdotal reports from herpetologists, land managers and Traditional Owners. We cross-referenced these reports with historical records obtained from the Atlas of Living Australia database (accessed 2017 and 2018).

We used two methods to determine the presence/absence and arrival date of cane toads to our 45 sites. Firstly, monitoring efforts of cane toads’ western and southern advance have increased over the last 30 years, and precise arrival dates were known for 19 of the most recently invaded sites. Based on the known distribution of toads we selected 11 toad-free sites presumed to be beyond the current known invasion distribution, and conducted extensive surveys (both prior to and during our experimental protocol) to confirm toad absence. However, the 15 sites in Queensland with the longest periods of toad occupation pre-date studies of the ecological impact of cane toads in Australia, and the precise timing of toad colonisation was unknown.

Accordingly, we used ArcGIS 10.5 (ESRI, Redlands, CA) to estimate the arrival dates of toads at these long-invaded sites. We retrieved historical cane toad occurrence records from the Atlas of Living Australia database (https://www.ala.org.au - accessed 2017) and arrived at a conservative estimate of the date of cane toad arrival by creating a 20 km radial buffer zone around each site and extracting the earliest occurrence record within each buffer zone. These records were cross-referenced with anecdotal historical accounts where available to confirm accuracy.

Sites along transects were categorised by ‘toad invasion-stage’ (i.e., the number of years since toads had first arrived in an area). To ensure our categories were biologically relevant for both varanid species, we calibrated invasion-stage categories by ‘number of varanid generations’ (rather than ‘years’); differences in generation time affect the opportunity for a varanid population to recover from cane toad arrival. We based estimates of the generation time of each goanna species on the shortest known period to reach sexual maturity: lace monitors– 2 years [46]; yellow-spotted monitors– 1 year (G. Ward-Fear, unpublished data). Thus, our sites were classified as “uninvaded” (toad free), “recently invaded” (1–12 goanna generations), “mid-term invaded” (13–29 goanna generations), and “long-term invaded” (30–80 goanna generations). These categories were chosen to provide approximately similar sample sizes within each invasion-history group.

### Survey protocol

We used a combination of standardised active searches and camera trapping at bait stations to estimate the relative abundance of varanids, small mammals (adult mass < 250 g), and cane toads at each site. All surveys were conducted by a single observer (LP). Survey effort was standardised to 1-h surveys in the morning, afternoon and night for five days at each site (total 15 surveys), with surveys timed to coincide with peak activity of the local varanid species.

For east coast sites, each survey consisted of a 15-min active search on foot around focal campsites (areas to which lace monitors are attracted) [16], and a 45-min search along a 5-km transect in a vehicle (paved and unpaved roads were used for transects). Morning surveys commenced from 0900 to 1200 h, afternoon surveys from 1200 to 1800 h, and nocturnal surveys from 1800 to 0100 h. In tropical Australia, yellow-spotted monitors are most active on relatively cool mornings [33]. Accordingly, at the twenty-four sites in our northern transect, morning surveys consisted of a 1-h active search on foot along a 2 km transect adjacent to focal wetlands, lakes, rivers, creeks, billabongs and floodplains (areas frequented by yellow-spotted monitors) ^33^. In the afternoon and evening we surveyed the same target areas on foot for 30 min, then spent 30 min surveying a 5-km transect through adjacent habitat by vehicle (along paved and unpaved roads). Morning searches commenced 30 min after sunrise, afternoon searches started 3 h before sunset, and night searches 30 min after sunset. For both east coast and tropical sites, we recorded varanids, small mammals and cane toads encountered on surveys with mobile application software [47].

In addition to the active searches, we deployed eight bait stations at each site, monitored with motion-activated cameras (Scoutguard SG560) to provide an additional measure of the relative abundance of varanids, small mammals and cane toads. For east-coast sites, bait stations and passive infrared motion sensing cameras were deployed at each site in two grids. Each grid consisted of four cameras (each was spaced 100 m apart) for a total of eight bait stations and cameras per site. One grid was placed in bushland adjacent to the focal campground and a second grid established in bushland 2 km away. Bait stations were deployed for 48 hours (total 16 camera days/nights per site) and consisted of one non-consumable chicken neck (deployed in a PVC ventilated canister to prevent predator access). An additional consumable chicken egg was placed at half of the bait stations at 13/21 sites for a concurrently run experiment.

In the wet/dry tropics, eight cameras were deployed along the walked transect in focal habitat, with adjacent cameras separated by at least 100 m. Eighty grams of non-consumable sardines in oil were placed in the PVC canister, a method suitable for attracting varanids and small mammals in northern Australia [48]. A cracked chicken egg was placed at the base of the bait station, and a single consumable sardine and cane toad leg (collected as road-kill, washed, frozen and thawed prior to deployment) were placed randomly 30 cm to either side of the egg and covered with a plastic lid (20 x 27 cm) with mesh window (for a concurrently run behavioural experiment). We predicted *a priori* that yellow-spotted monitors would be more difficult to detect by active search surveys compared with lace monitors. Accordingly, cameras were deployed in sites with yellow-spotted monitors for 120 h (total 40 camera days/nights per site).

Cameras were programmed to capture 1 min of video footage when triggered. Based on pilot studies, we used a 30-min event period to quantify relative abundances of varanids from camera footage. Events were considered independent if they were separated by 30 min without a trigger during the intervening period. For small mammals or toads (which spent less time at the bait station, but often with more than one individual), once an animal was detected on video we analysed the next 30 min of videos and used the count with the highest number of individuals within that timeframe to estimate the relative abundance of that species during that event.

For logistical reasons, sites were surveyed concurrently in groups of one to three. The sequence in which grouped sites were surveyed was randomised to minimise the effect of season, longitude and latitude. We also randomised the order that sites within groups were surveyed, to reduce time-of-sampling effects. To reduce seasonal biases, the five days of sampling at each site were conducted across at least two different times of the season (i.e., early and mid wet-season).

### Statistical analyses

All linear model statistical tests were performed in R (v3.6.0) [49]. We used generalised linear mixed models (GLMM) with a Poisson distribution and log-link function within the “lme4” v.1.1–23 package [50]. Our dependent variables were counts of monitor lizards, toads, and mammals from combined totals of each day’s surveys and camera captures (105 survey and 42 camera replicates for the east coast, and 120 survey and 120 camera replicates for the tropical transect). For all models, we included survey type (active search or camera) to account for the different survey methodologies, and site was included as a random factor to account for multiple surveys per site. Following significant main effects, we used estimated marginal means contrasts to explore comparisons between invasion categories within the “emmeans” package [51]. To test for spatial autocorrelation between sites, we incorporated model residuals from the relative abundance estimates of each varanid species in Moran’s I tests using ArcGIS 10.5 (ESRI, Redlands, CA). Model residuals were not significant either for lace monitors or for yellow-spotted monitors (Moran’s I = 0.014, Z = -1.55, p = 0.12; Moran’s I = -0.010, Z = -0.45, p = 0.65, respectively).

#### Abundance of varanid lizards

To assess correlates (and thus, potential determinants) of the relative abundance of varanids, we ran GLMMs with all combinations of factors within the “MuMIn” package [52] on the overall datasets to rank alternative models. Our models included (i) time since toad invasion (categorical variable with four levels; uninvaded, recently invaded, mid-term invaded, and long-term invaded) to test the effect of invasion history on the number of lace monitors or yellow-spotted monitors encountered per day. Plausibly, our counts of varanid lizards also might be affected by biotic and abiotic factors that could influence the abundance of animals, or modify our ability to detect the animals even if they are present. Thus, we also included (ii) mean rainfall during the wet season [Oct-May] (as environmental productivity is driven by seasonal rainfall) [53], (iii) the relative abundance of cane toads (a proxy for risk of lethal toxic ingestion), (iv) the relative abundance of small mammals (a proxy for availability of prey), and (v) the interaction between mammal abundance and toad abundance.

Additionally, we might fail to see lizards because of dense vegetation (concealing animals) or cool weather (reducing lizard activity). We thus included the (vi) daily maximum temperature and (vii) mean ground cover (percentage cover > 30 cm high in the area visible to cameras beyond each cleared bait station) for each survey day per site (from the closest Bureau of Meteorology weather station) to account for variation in these factors between surveys and sites. Finally, preliminary modelling showed that (viii) survey type was a significant influence on monitor numbers. We therefore included survey type to account for potential variability in detection between our two survey methods (active search vs. motion-sensing cameras), but as this factor does not relate to a specific hypothesis, we do not report on it further in the results. We used Akaike’s Information Criterion (AICc) scores to identify the most parsimonious models with the best fit to the data (lower AICc values indicate models with greater support) [54]. We then calculated the relative variable importance (RVI) for all factors to rank the importance of each variable in predicting the abundance of lizards [55].

#### Abundance of cane toads

First, we used a GLMM to examine if toad numbers at toad-present sites differed between east coast and tropical transects. Due to absence of toads in the uninvaded category, we then tested the effect of invasion stage (three levels; recently invaded, mid-term invaded, and long-term invaded) on the relative abundance of cane toads present along the east coast transect and through the tropical transect using GLMMs.

#### Abundance of small mammals

We collated counts of small mammals for each site, as a proxy for prey availability, and used a GLMM to ask if the average relative abundance of small mammals differed between east coast sites versus tropical transect sites. To compare the relative abundances of small mammals across invasion categories, we used GLMM to examine the effect of invasion stage on the number of small mammals within each transect.

### Ethical statement

All procedures were approved by the University of Sydney ethics committee (approval 2017/1202) and were carried out in accordance with relevant guidelines and regulations under licence from state and federal wildlife agencies.

## Results

### Abundance of varanid lizards

**Fig 3:**
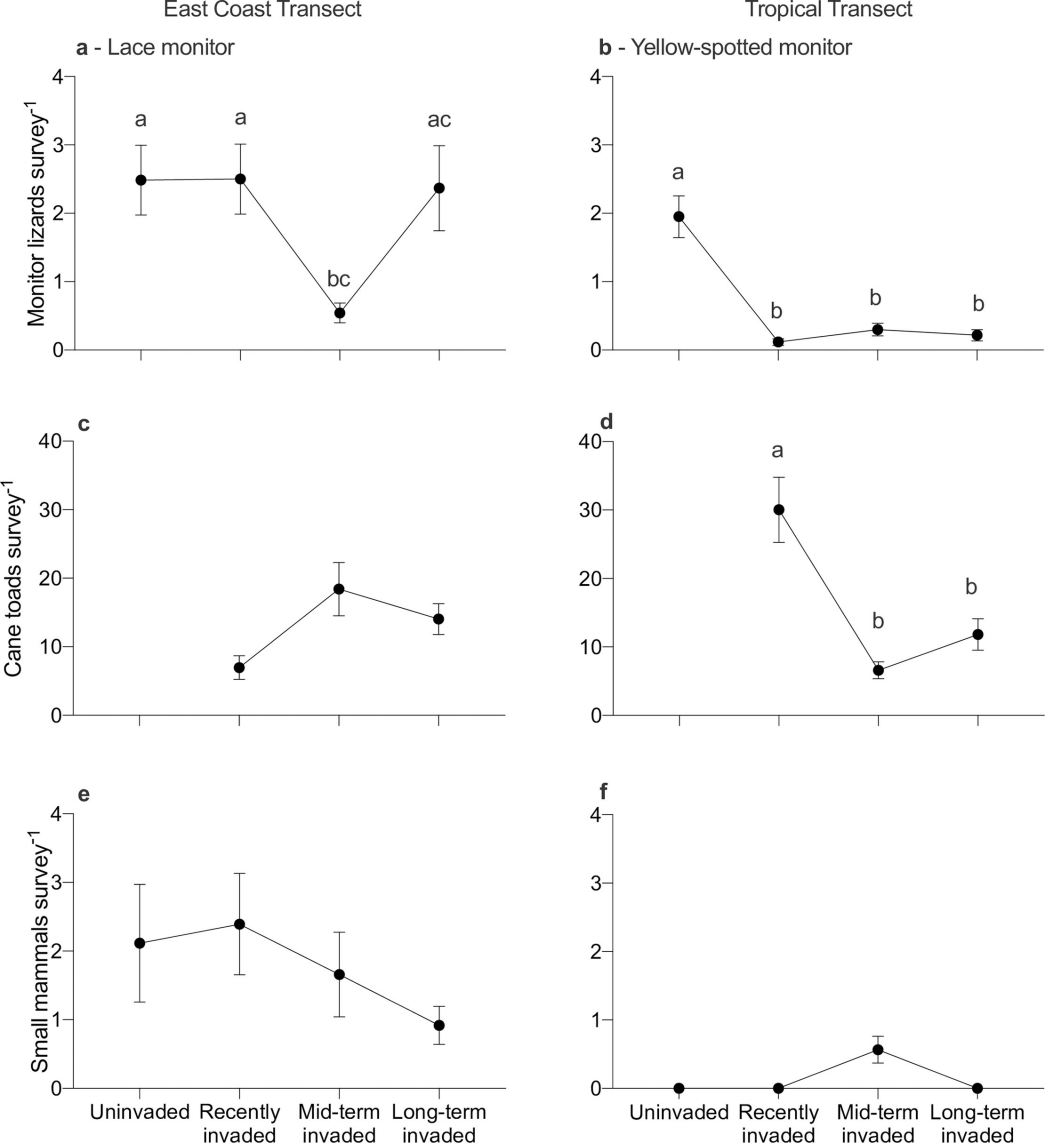
**Numbers of animals detected per day based on active searches and remote-sensing cameras at 21 sites along the east coast of Australia (left panels) and 24 sites across the tropical transect (right panels).** Top panels display the mean (± se) number of the varanid lizards (**a**) lace monitors (*Varanus varius*) and (**b**) yellow-spotted monitors (*V*. *panoptes*), middle panels (**c, d**) represent the mean (± se) number of cane toads (*Rhinella marina*) recorded, and lower panels (**e, f**) show the mean (± se) number of small mammals (< 250 g) detected. The invasion categories are based on the number of varanid-generations for which cane toads have been present in the landscape (uninvaded–toads not present; recently invaded–toads present for 1–12 varanid generations; mid-term invaded–toads present for 13–29 varanid generations; long-term invaded–toads present for 30–80 varanid generations). Following a significant main effect of invasion stage, we ran post-hoc estimated marginal means contrasts to test for significant differences between invasion stages. Cases with p < 0.05 are indicated by contrasting letters.

Along the eastern coast of Australia, the relative abundance of lace monitors did not change significantly when toads arrived, although numbers were lower at the mid-term invaded sites than elsewhere (Table 1, Fig 3A). Although our model selection identified that invasion stage featured in 7 highly ranked models, a low RVI of 0.43 for this variable suggests that invasion stage is not an important predictor of the abundance of lace monitors. The factor that explained the most variance in counts of lace monitors was the number of small mammals (i.e., potential prey), with 7 of the equal top-ranked models (Δ AIC <2) including this term (RVI = 0.50). Neither the number of toads per site (6 models; RVI = 0.43), nor the interaction between toads and mammals (1 model; RVI = 0.09) were strongly supported in our models. Likewise, vegetation (2 models; RVI = 0.30), rainfall (3 models; RVI = 0.32), and temperature (4 models; RVI = 0.33) did not strongly predict the abundance of lace monitors.

**Table 1:** Akaike’s information criterion (AICc) scores were used to rank candidate models testing the effect of invasion stage (uninvaded, recently invaded, mid-term invaded and long-term invaded), mammals (counts), toads (counts), the interaction between mammals and toads, temperature, vegetation, and rainfall on the relative abundance of lace monitors (*Varanus varius*) and yellow-spotted monitors (*Varanus panoptes*) at 45 sites in Australia.

*Varanus varius*											
Invasion stage	Mammals	Toads	Mammals x toads	Max temperature	Vegetation	Rainfall	df	logLik	AICc	Δ AICc	*w*
+	-	-	-	-	-	-	6	-297.921	608.442	0.000	0.050
-	0.078	-	-	-	-	-	4	-300.086	608.454	0.012	0.049
-	-	-0.023	-	-	-	-	4	-300.143	608.568	0.126	0.047
+	0.061	-	-	-	-	-	7	-296.965	608.736	0.294	0.043
-	-	-	-	-	-	-	3	-301.460	609.089	0.647	0.036
-	0.079	-	-	0.038	-	-	5	-299.534	609.493	1.051	0.029
+	-	-	-	-	-0.019	-	7	-297.395	609.597	1.155	0.028
-	-	-0.021	-	-	-	0.001	5	-299.596	609.617	1.175	0.028
-	0.052	-0.015	-	-	-	-	5	-299.638	609.701	1.259	0.026
-	-	-	-	-	-	0.001	4	-300.716	609.714	1.272	0.026
-	-	-0.023	-	0.034	-	-	5	-299.692	609.809	1.367	0.025
+	0.061	-	-	-	-0.019	-	8	-296.390	609.824	1.382	0.025
+	0.097	-	-	-	-	-0.001	8	-296.461	609.966	1.524	0.023
-	-	-	-	0.038	-	-	4	-300.910	610.102	1.660	0.022
+	-	-	-	0.024	-	-	7	-297.698	610.202	1.760	0.021
-	0.027	-0.026	0.006	-	-	-	6	-298.844	610.287	1.846	0.020
+	-	-0.007	-	-	-	-	7	-297.816	610.437	1.995	0.018
-	-	-	-	-	-	-	2	-307.927	619.937	11.495	0.000
*Varanus panoptes*											
Invasion stage	Mammals	Toads	Mammals x toads	Max temperature	Vegetation	Rainfall	df	logLik	AICc	Δ AICc	*w*
+	-	-	-	0.151	-	0.003	8	-189.258	395.139	0.000	0.201
+	-	-	-	0.137	-	-	7	-191.095	396.672	1.533	0.093
+	-	-0.010	-	0.149	-	0.003	9	-189.045	396.873	1.734	0.084
+	-	-	-	0.140	0.020	-	8	-190.129	396.880	1.742	0.084
+	-0.304	-	-	0.150	-	0.003	9	-189.054	396.890	1.751	0.084
+	-	-	-	0.151	0.009	0.003	9	-189.110	397.002	1.863	0.079
-	-	-0.045	-	0.166	-	-	5	-195.066	400.389	5.250	0.015
+	-	-	-	-	-	-	6	-194.974	402.309	7.170	0.006
-	-	-	-	-	-	-	2	-244.664	493.378	98.240	0.000

All models included survey type to account for the different survey techniques (active search and motion-sensing cameras) but are not presented in the table. We present the equal top-ranked models with a ΔAICc < 2 and the null model for each species. For yellow-spotted monitors, we present the highest-ranked models that exclude invasion and temperature.

In contrast, the relative abundance of yellow-spotted monitors in tropical Australia declined as soon as toads arrived, and remained low thereafter (Table 1, Fig 3B). Our model selection identified invasion stage as an important factor, with an RVI of 0.96; the top-ranked model including invasion stage outranked the top-ranked model without that factor by 5.25 AICc units. Similarly, ambient temperature (RVI = 0.96) was an important predictor of the abundance of yellow-spotted monitors with the top-ranked model including that factor 7.17 AICc units higher than the top-ranked model without temperature. There was also support for rainfall as a significant predictor of the numbers of yellow-spotted monitors (RVI = 0.60). In contrast, the relative abundance of yellow-spotted monitors was not strongly predicted by the number of small mammals (RVI = 0.30), the number of toads or vegetation density (both factors with RVI = 0.34), nor the interaction between the number of toads and mammals (RVI = 0.03).

### Abundance of cane toads

Overall, the average number of cane toads recorded did not differ significantly between the east coast versus tropical transects (toad-present sites only, GLMM; Z = 1.61, p = 0.11). Within toad-present sites on the east coast, there was no significant difference in the mean numbers of toads among invasion-time categories (Table 2, Fig 3C). In contrast, the significant effect for toad relative abundance along the tropical transect (Table 2, Fig 3D) was driven by a spike in toad numbers at recently-invaded sites.

**Table 2:** GLMM outputs for models testing relative abundance of cane toads (*Rhinella marina*) as a function of time since cane toads invaded.

	East coast					Wet/dry tropics				
	Estimate	Std. Error	z value	Pr(>|z|)		Estimate	Std. Error	z value	Pr(>|z|)	
(Intercept)	1.634	0.631	2.588	**0.010**	**	3.740	0.376	9.944	**< 0.0001**	***
Survey type	-4.619	0.408	-11.320	**< 0.0001**	***	-1.669	0.046	-36.278	**< 0.0001**	***
Mid-term invaded	1.034	0.842	1.227	0.220		-1.269	0.469	-2.703	**0.007**	**
Long-term invaded	0.908	0.788	1.153	0.249		-1.167	0.456	-2.557	**0.011**	*

Mid-term and long-term invaded categories are tested against the reference “recently invaded” category. Cases with p-values < 0.05 are highlighted in bold.

### Abundance of small mammals

Small mammals were far less abundant along the tropical transect (mean = 0.14 ± 0.05 se per survey) than the east-coast transect (mean = 1.7 ± 0.3 se per survey; Z = -5.30, p < 0.0001). The relationship between toad invasion phase and relative abundance of small mammals also differed between the two transects. On the east-coast transect, mean numbers of small mammals did not differ significantly between sites with versus without toads (Z = -0.50, p = 0.62), or with toads established for differing periods of time (Table 3, Fig 3E). Small mammals were scarce right across the tropics, with no mammals detected in three of the four invasion stages (Fig 3D). No analysis was run on these data.

**Table 3:** GLMM outputs for models testing relative abundance of small mammals (mass < 250 g) as a function of time since cane toads invaded.

	East coast				
	Estimate	Std. Error	z value	Pr(>|z|)	
(Intercept)	-1.367	0.648	-2.109	**0.035**	**
Survey type	2.546	0.173	14.721	**< 0.0001**	***
Recently invaded	0.654	0.923	0.708	0.479	
Mid-term invaded	-0.398	0.898	-0.443	0.658	
Long-term invaded	-1.112	0.850	-1.308	0.191	

Recently-invaded, mid-term-invaded and long-term invaded categories are tested against the reference “uninvaded” category. Cases with p-values < 0.05 are highlighted in bold. Analyses on data for mammals from the wet/dry tropics was not analysed due to absence of mammals in three invasion-stage categories.

## Discussion

The two varanid lizard species that we studied differed dramatically in their numerical response to invasion by cane toads. The east-coast woodland species (*V*. *varius*) showed no significant impact following the arrival of toads, whereas the tropical floodplain species (*V*. *panoptes*) exhibited a dramatic and immediate decline in numbers, with few signs of recovery at sites that have been occupied by toads for up to 80 years (Fig 3A and 3B). The spatial variation in our counts of lizards was not correlated with factors (dense vegetation and cool weather) that might plausibly affect detectability, suggesting that the two varanid taxa have indeed responded very differently to the arrival of cane toads. Below, we consider potential explanations for this marked disparity in responses.

The greater vulnerability of yellow-spotted monitors to cane toad invasion may reflect their preference for waterside habitats, resulting in a high consumption of aquatic prey [33]. That association with riparian zones must bring foraging varanids into frequent contact with cane toads, that remain in moist sites to avoid desiccation during the long tropical dry-season [56]. Foraging within areas that contain high densities of toads is a consistent trait of taxa impacted by these invasive anurans [57]. Especially near the end of the dry-season, when waterbodies shrink, both yellow-spotted monitors and cane toads are concentrated in the same places within an increasingly parched landscape, particularly at the invasion front where toad density is highest. Such encounters may be dangerous for goannas, even for individuals that have learnt taste aversion to toads [44]. A single mistake can be fatal. In keeping with that interpretation, Llewelyn et al. [21] reported a single case of a yellow-spotted monitor from a long-colonised population eating a cane toad; and we witnessed a similar case (a yellow-spotted monitor consuming a road-killed toad) in the same region during the present study.

The location and timing of resource availability can greatly impact an animal’s vulnerability to threatening ecological processes [58]. In this case, the invasive (toxic) cane toad may be one of the few large prey items available to yellow-spotted monitors at some times of year–especially, since the decline of small mammals across most of the range of this varanid species [2, 59–61]. In keeping with dietary analyses that report frequent consumption of small mammals by large varanids [28, 33, 35, 38], we detected a positive correlation between the relative abundances of small mammals and of lace monitors, but no correlation with yellow-spotted monitors. The pattern for yellow-spotted monitors reflects the virtual absence of records of small mammals all across our transect in northern Australia.

The broadscale decline of small mammal populations across tropical Australia over recent years (to near-extinction in many areas [59]) essentially removed that potential high-protein prey base (see Fig 3F) and may have encouraged yellow-spotted monitors to forage primarily in the times and places where they are most likely to encounter toxic toads. Broadly, the sites where yellow-spotted monitors have persisted despite the presence of cane toads appear to contain unusually high availability of alternative prey. Thus, for example, yellow-spotted monitors remain common (1) at a site on the Adelaide River floodplain, Northern Territory, where a commercial fish farm provides abundant food (P. Fisher, pers. comm.); (2) at Wreck Rock, on the Queensland coast, and Coburg Peninsula on the Northern Territory coast, where the lizards forage along beaches for sea-turtle eggs [62] (G. Ward-Fear pers. obs.); and (3) at Townsville Common, north-east Queensland, where abundant keelback snakes (*Tropidonophis mairii*) provide an alternative food resource (L. Pettit, unpublished data). This anecdotal correlation between varanid abundance and food availability needs to be explored with more extensive surveys, that document the food-resource base as well as varanid numbers.

Why, then, are yellow-spotted monitors abundant in sites within north-western Australia where cane toads have not yet arrived, but where our surveys detected few small mammals (Fig 3F)? Part of the answer may lie in shortcomings of our sampling regime. For example, mammal abundance may vary seasonally and differ among years, depending upon rainfall patterns [35], so that we may have failed to detect small mammals even when they were episodically abundant. Other impacts of toad invasion might play a role also. The near-extirpation of large varanids induces mesopredator release, with potential impacts on smaller prey [6]. Hence, invasive toads may indirectly depress prey availability for varanids, via trophic cascades–exacerbating the dramatic reduction in prey resources caused by the disappearance of small mammals across most of tropical Australia [2, 61].

In summary, we suggest that yellow-spotted monitors face a perfect storm when cane toads arrive. Yellow-spotted monitors first encounter toads at the invasion front, where toads reach extremely high population densities. These giant lizards forage primarily in places where cane toads are common (especially seasonally, at a time when few alternative prey are present). The availability of alternative prey may have been reduced by the decline of small mammals, likely due to feral predators (especially cats), agricultural practices, and modified fire regimes [59]. Also, the latter factors may have affected populations of varanid lizards, independent of the impacts of cane toads.

Given their superficial similarities to yellow-spotted monitors, why were lace monitors so much more resilient to toad invasion? These east-coast lizards are distributed in forested areas, often far from water, and forage in arboreal as well as terrestrial habitats [31, 63]. As a result, they consume fewer aquatic prey (including frogs) than do *V*. *panoptes* (reviewed in [38]). Cane toads are rarely arboreal [64], decreasing encounter rates of toads with lace monitors (especially smaller individuals) [65], which might otherwise be at greater risk from ingesting a toad. Relatively aseasonal rainfall over much of the range of lace monitors also may eliminate a dangerous scenario encountered by yellow-spotted monitors: the concentration of both predators and cane toads around shrinking waterbodies during the dry-season. Lastly, small mammals remain common in many forested habitats along the eastern coast of Australia [66] (and see Fig 3E and 3F), allowing lace monitors to forage in drier habitats where toads are scarce.

Overall, our results paint a more optimistic picture for lace monitors than was suggested by earlier studies [16, 30]. Plausibly, the mortality caused by toad invasion is rapidly balanced by enhanced rates of survival of more cautious younger lizards [30], and perhaps by immigration of conspecifics from surrounding forests into campground areas which offer rich food subsidies [67]. If so, decreases in overall numbers of varanids at campgrounds (the places where we sampled) likely are relatively brief. The numerical decline of lace monitors in the mid-term invasion stage is puzzling, and is unlikely to correspond to toad presence. Lace monitors in areas long colonised by toads avoid eating the toxic anurans, suggesting that toad abundance should have little impact on varanid vulnerability [68].

Importantly, our analyses suggest that the impact of an invasive species (the cane toad) on a native predator (the yellow-spotted monitor) was exacerbated by a reduction in prey availability, likely due to other anthropogenic changes to the landscape. For example, predation by cats has devastated populations of small mammals across much of tropical Australia [59]. This example highlights the interactive effects of multiple ecosystem stressors in driving population declines of native species, and in preventing recovery after population collapse [69, 70]. If we are to address the root causes of ecosystem decline across tropical Australia, we need to simultaneously consider the multiple challenges imposed by anthropogenic processes.
